# Sequencing viral genomes from a single isolated plaque

**DOI:** 10.1186/1743-422X-10-181

**Published:** 2013-06-06

**Authors:** Jessica DePew, Bin Zhou, Jamison M McCorrison, David E Wentworth, Janaki Purushe, Galina Koroleva, Derrick E Fouts

**Affiliations:** 1 Department of Genomic Medicine, The J. Craig Venter Institute (JCVI), 9704 Medical Center Drive, Rockville, MD 20850, USA; 2 Department of Infectious Disease, JCVI, 9704 Medical Center Drive, Rockville, MD, 20850, USA; 3 Informatics Core Services, JCVI, 9704 Medical Center Drive, Rockville, MD, 20850, USA

**Keywords:** SISPA, Sequencing, Virus, Single plaques, Bacteriophage

## Abstract

**Background:**

Whole genome sequencing of viruses and bacteriophages is often hindered because of the need for large quantities of genomic material. A method is described that combines single plaque sequencing with an optimization of Sequence Independent Single Primer Amplification (SISPA). This method can be used for *de novo* whole genome next-generation sequencing of any cultivable virus without the need for large-scale production of viral stocks or viral purification using centrifugal techniques.

**Methods:**

A single viral plaque of a variant of the 2009 pandemic H1N1 human Influenza A virus was isolated and amplified using the optimized SISPA protocol. The sensitivity of the SISPA protocol presented here was tested with bacteriophage F_HA0480sp/Pa1651 DNA. The amplified products were sequenced with 454 and Illumina HiSeq platforms. Mapping and *de novo* assemblies were performed to analyze the quality of data produced from this optimized method.

**Results:**

Analysis of the sequence data demonstrated that from a single viral plaque of Influenza A, a mapping assembly with 3590-fold average coverage representing 100% of the genome could be produced. The *de novo* assembled data produced contigs with 30-fold average sequence coverage, representing 96.5% of the genome. Using only 10 pg of starting DNA from bacteriophage F_HA0480sp/Pa1651 in the SISPA protocol resulted in sequencing data that gave a mapping assembly with 3488-fold average sequence coverage, representing 99.9% of the reference and a *de novo* assembly with 45-fold average sequence coverage, representing 98.1% of the genome.

**Conclusions:**

The optimized SISPA protocol presented here produces amplified product that when sequenced will give high quality data that can be used for *de novo* assembly. The protocol requires only a single viral plaque or as little as 10 pg of DNA template, which will facilitate rapid identification of viruses during an outbreak and viruses that are difficult to propagate.

## Background

During a viral outbreak, it is highly desirable to rapidly determine the identity of the causative agent through whole genome sequencing. Genome sequencing of novel viruses and bacteriophages (phages) is often difficult and time consuming due to the need to grow large-scale, high titer lysates in order to obtain a sufficient quantity of viral nucleic acids for whole genome sequencing. Though metagenomic techniques have been utilized to sequence uncultured viruses and phages from the environment [1,2] and the human gut [3-5], the genome sequence of any single virus in these samples is typically incomplete. These techniques also require nanogram or microgram quantities of nucleic acid for library construction and require highly purified viral particles to prevent sequencing of contaminating host genomes. Furthermore, it is difficult to know the host of viruses taken from a metagenomic study unless the virus is cultivated on a suitable host.

Methods have been described for the direct Sanger sequencing of λ and M13 library clones from single plaques [6,7], but are unsuitable for *de novo* whole genome sequencing of novel viruses because they were designed to sequence foreign DNA cloned into phage vectors using phage specific oligonucleotide primers rather than complete phage genomes primed using random primers. The objective of this study was to optimize a method that combines single plaque sequencing with an optimized random-primed amplification method [8] that can be used for *de novo* genome sequencing of any cultivable virus without the need for large-scale production of viral stocks or viral purification using ultracentrifugal techniques.

## Methods

### General

All enzymatic reactions were prepared with sterile water that was deionized using a Milli-Q^®^ water purification system. For RNA work, all reagents were made with DEPC-treated sterile deionized water. Benzonase^®^ was obtained from Sigma Chemical Company. RNases A and T1 were obtained from Ambion. EMEM, BSA fraction V, antibiotic-antimycotic, RNaseOUT™ and SuperScript^®^ III Reverse Transcriptase (SSIIIRT) were obtained from Invitrogen. Tosylsulfonyl phenylalanyl chloromethyl ketone (TPCK)-treated trypsin was purchased from Worthington Biochemical Corporation (Lakewood, NJ). BioMix™ Red DNA polymerase was obtained from Bioline. The RNeasy Mini and QIAquick Gel Extraction kits were purchased from Qiagen. Polyethylene glycol (PEG) 8000 was obtained from USB Corporation. All other enzymes and dNTPs were obtained from New England Biolabs. All reagents used were of molecular biology grade or higher.

### Influenza virus plaque growth

To obtain a single plaque of a mouse-adapted variant (NY1682-MAP7) of a 2009 H1N1 pandemic Influenza A virus [9], a monolayer of MDCK cells [ATCC CCL-34] in a six-well plate was infected with serially diluted virus for 1 h and covered with an overlay containing 1% agarose, 1X EMEM, 0.3% BSA fraction V, 1% antibiotic-antimycotic, and 2 μg/ml TPCK-treated trypsin. After two days of incubation at 37°C, a single plaque was picked using a sterile Pasteur pipette. The plug was incubated overnight in SM buffer (0.01% gelatin, 250 mM NaCl, 8.5 mM MgSO_4_, 50 mM Tris–HCl, pH 7.5) to release the viral particles from the agarose.

### Nucleic acid extraction

After overnight incubation, the viral plug was filtered through a 0.45 μm syringe filter to remove host cells. The filtrate was treated with 125 U Benzonase, 10 U DNase I, 50 U RNase A and 200 U RNase T1 at 37°C for 1 h. Nucleases were deactivated by bringing the concentrations of both EDTA and EGTA up to 50 mM. Influenza viral RNA was purified using the RNeasy Mini Kit from Qiagen following the manufacturer’s instructions. The RNA was eluted in 25 μl of RNase-free DEPC-treated water.

### Modified SISPA protocol

Bacteriophage F_HA0480sp/Pa1651 Klenow reactions were performed in duplicate to increase the coverage of the target genome [10]. Template DNA was added so that 10 pg of DNA was added in a 2 μl volume, and 0.5 μl of 50% DMSO was added to aid in denaturing. Templates were incubated at 95°C for 5 min on a thermal cycler followed immediately with snap cooling on ice. After cooling for 5 min, 1 μl of 100 μM barcoded random hexamer primer FR20RV-N [8] (5′ GCC GGA GCT CTG CAG ATA TCN NNN NN 3′) or BC081N (5′ CGA GAG ATA CTG TAC TAG AGC GNN NNN N 3′) for Illumina or 454 sequencing, respectively, was added to each reaction. To allow optimal binding of the random primers, the reactions were incubated with a 1°C per min ramp from 4°C to 37°C on the thermal cycler. Random-primed amplification was achieved using 1.5 U exo- Klenow fragment, 1× NEB Buffer 2 and 0.2 mM dNTPs in 5 μl reactions incubated at 37°C for 1 h. A second round of amplification was performed with the addition of 2.5 U of exo- Klenow fragment incubated at 37°C for 1 h. The Klenow reaction was terminated by heat inactivation at 75°C for 15 min.

Influenza A virus amplification reactions were also performed in duplicate. The RNA template was split into two aliquots for first strand synthesis, and 2 μl of 100 μM barcoded random hexamer primer BC391N (5′ CGT GAC TAT CTC GCG AGT ACG ANN NNN N 3′), 1 μL of solution containing 10 mM of each dNTP and 0.6 μL of 10% DMSO were added to each. Final volumes were brought up to 10 μL with DEPC-treated water and samples were incubated at 96°C for 5 min, then snap-cooled on ice. 4 μl 5× First Strand Buffer (Invitrogen), 2 μl 0.1 M DTT, 0.2 μl RNaseOUT (40 U/μL), 0.5 μl SSIIIRT (200 U/μl), and 3.3 μl DEPC-treated water were added to each sample. Reverse transcription occurred on the thermal cycler at 25°C for 10 min followed by 50°C for 50 min, and finished by incubation at 85°C for 10 min before being snap-cooled on ice. To destroy the RNA template, the reaction was then treated with 5 U RNase H and incubated at 37°C for 20 min followed by heat inactivation at 85°C for 10 min. A second round of amplification was performed with the addition of 2.5 U exo- Klenow fragment and incubation at 37°C for 1 h followed by heat inactivation at 75°C for 15 min.

The cleanup of Klenow reactions involved removal of primers and short fragments by diluting with 20 μl of water and mixing with 10 μl of 30% PEG 8000 with 5 mM MgCl_2_[11] to adjust the final concentration of PEG and MgCl_2_ to 8.7% and 1.4 mM, respectively. Mixtures were then incubated on ice for 15 min and then centrifuged at 16100 × g at 4°C for 30 min. The supernatant from each reaction was removed and pellets were reconstituted with 20 μl of water. Single-stranded fragments were removed by treatment with 20 U of Exonuclease I in 1 × Exonuclease I buffer at 37°C for 30 min. Exonuclease I was then heat inactivated at 80°C for 20 min.

### Amplification of double-stranded SISPA products

Duplicate Klenow reactions were pooled after cleanup. PCR reactions contained 5 μl of cleaned Klenow product, 400 nM of barcoded primer (lacking the 3′ random hexamer) and 25 μl of BioMix Red in a total volume of 50 μl. PCR conditions included an initial denaturation step at 98°C for 30 s followed by 35 cycles of (98°C for 10 s, 54°C for 20 s, 72°C for 45 s). A final extension at 72°C for 5 min completed the PCR. The completed reactions were reduced to half of the starting volume in a Thermo Savant DNA 120 SpeedVac and then the entire reaction was loaded onto a 1.2% agarose gel and stained with ethidium bromide. Smears between 300 and 850 bp in size were extracted from the agarose gel using a QIAquick Gel Extraction Kit following the manufacturer’s instructions. The DNA was eluted with 12.5 μl of TE buffer pre-warmed to 65°C.

### 454 sequencing

Viral genomes were sequenced with the 454 FLX Titanium platform. Library construction, emulsion PCR (emPCR), enrichment and 454 sequencing were performed by following the vendor’s standard protocols, with some modifications. Specifically, SISPA products were not sheared and entered the library preparation workflow at the standard adaptor ligation step. Quantitative PCR (qPCR) was used to accurately estimate the number of molecules needed for emPCR using a KAPA Biosystems Library Quantification Kit. A BioMek^®^ FX automation workstation was used to “break” the emulsions after emPCR and butanol was used to enable easier sample handling during the breaking process. The REM e (Robotic Enrichment Module) from Roche was used to automate the bead enrichment process in the pipeline.

### Illumina sequencing

Viral genomes were also sequenced with the Illumina HiSeq 2000 platform. Libraries were prepared following Illumina’s standard protocol, with a few exceptions. As with the 454 library construction procedure, SISPA products were not sheared and entered the library preparation workflow at the DNA end repair step. All cleanup steps were performed using Agencourt AMPure XP beads. The libraries were quantitated and quality controlled using the Agilent High Sensitivity DNA Kit and by qPCR using a KAPA Biosystems Library Quantification Kit. Cluster generation and sequencing were completed utilizing Illumina’s standard protocol.

### Sequence preprocessing

Sequences were *de novo* assembled using the Newbler GS *De Novo* Assembler version 2.6 (Roche Diagnostics Corp., Indianapolis, IN) after the following pre-processing steps: 1) removal of host contamination by mapping reads to a contaminant reference database (see below) using the CLC Assembly Cell’s long read reference mapper with a minimum query length of 40% and 95% identity; 2) k-mer normalization (i.e., read correction) followed by exact sequence deduplication using a partial run of ALLPATHS-LG [12]; 3) mask low complexity/highly repetitive regions using DUST [13]; 4) dynamically quality trim reads using CLC Assembly Cell, cutoff QV = 18 or 2 contiguous ambiguous bases; 5) post-trimming contaminant removal as in step 1; and 6) secondary barcode removal of partial SISPA adapter matches using CutAdapt [14]. For step 1, the contaminate database for filtering the influenza sample consisted of the human and canine genomes. For the phage sample, the contaminate database consisted of human genome and the genome of *Pseudomonas aeruginosa* strain PAO1 [GenBank:AE004091] (i.e., the host genome) with Phage_Finder [15] predicted prophage regions masked out.

### Sequence assembly and mapping

The resulting cleaned-up reads were assembled using Newbler GS *De Novo* Assembler with minimum fragment length of 40 to allow use of Illumina reads shortened by adapter trimming. For *de novo* assemblies, read coverage was reduced to uniform levels via cross-comparison of read median k-mer frequencies. Sequence reads or contigs were mapped using the high-throughput “Map Reads to Reference” software in CLC Workbench version 5.5.1 (http://www.clcbio.com) using default settings. Identification of single nucleotide polymorphisms (SNPs) in the *de novo* assembled contigs was performed using the “Probabilistic Variant Detection” software in CLC Workbench using default settings.

## Results

**Figure 1:**
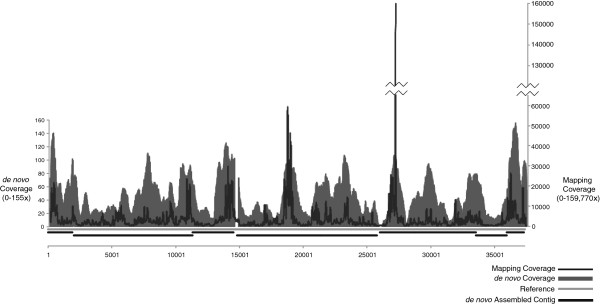
**Mapping and *de novo *assembly coverage sequencing results for the phage SISPA product from 10 pg of genomic DNA. **The black lines along the x-axis are the *de novo *assembled contigs mapped to the reference (light gray line across the x-axis). Coverage for the *de novo* contigs is shown with dark gray bars in the graph (left y-axis). The coverage from mapping the reads to the reference is represented with a black line (right y-axis).

The lowest concentration of template required for successful amplification using the modified SISPA protocol was determined by testing a serial dilution of a template phage whose complete genome sequence is known, bacteriophage F_HA0480sp/Pa1651 [GenBank:JN808773.1], which is 37,374 bp in length. Gel-purified SISPA product generated from 10 pg of purified bacteriophage F_HA0480sp/Pa1651 genomic DNA produced a total of 32,415 454 Titanium reads and 5,111,598 HiSeq reads. This small amount of starting material was sufficient to produce mapping assemblies with 3488-fold average sequence coverage, representing 99.9% of the reference and *de novo* assemblies with 45-fold average sequence coverage, representing 98.1% of the reference (Figure 1). When the *de novo* assembled contigs were compared to the reference there were no SNPs observed. The number of reads used for assembly was reduced after removing low levels of human contamination (0.05%), *Pseudomonas aeruginosa* host contaminantion (1.4%) and variable length SISPA adapter contamination (4.0%). In comparison, the *de novo* assembly result is more robust when more DNA (30 ng) was used in SISPA reactions, which resulted in one assembled contig used to generate the GenBank reference (data not shown); however, the majority of the genome sequence was obtained from as little as 10 pg of template DNA.

**Figure 2:**
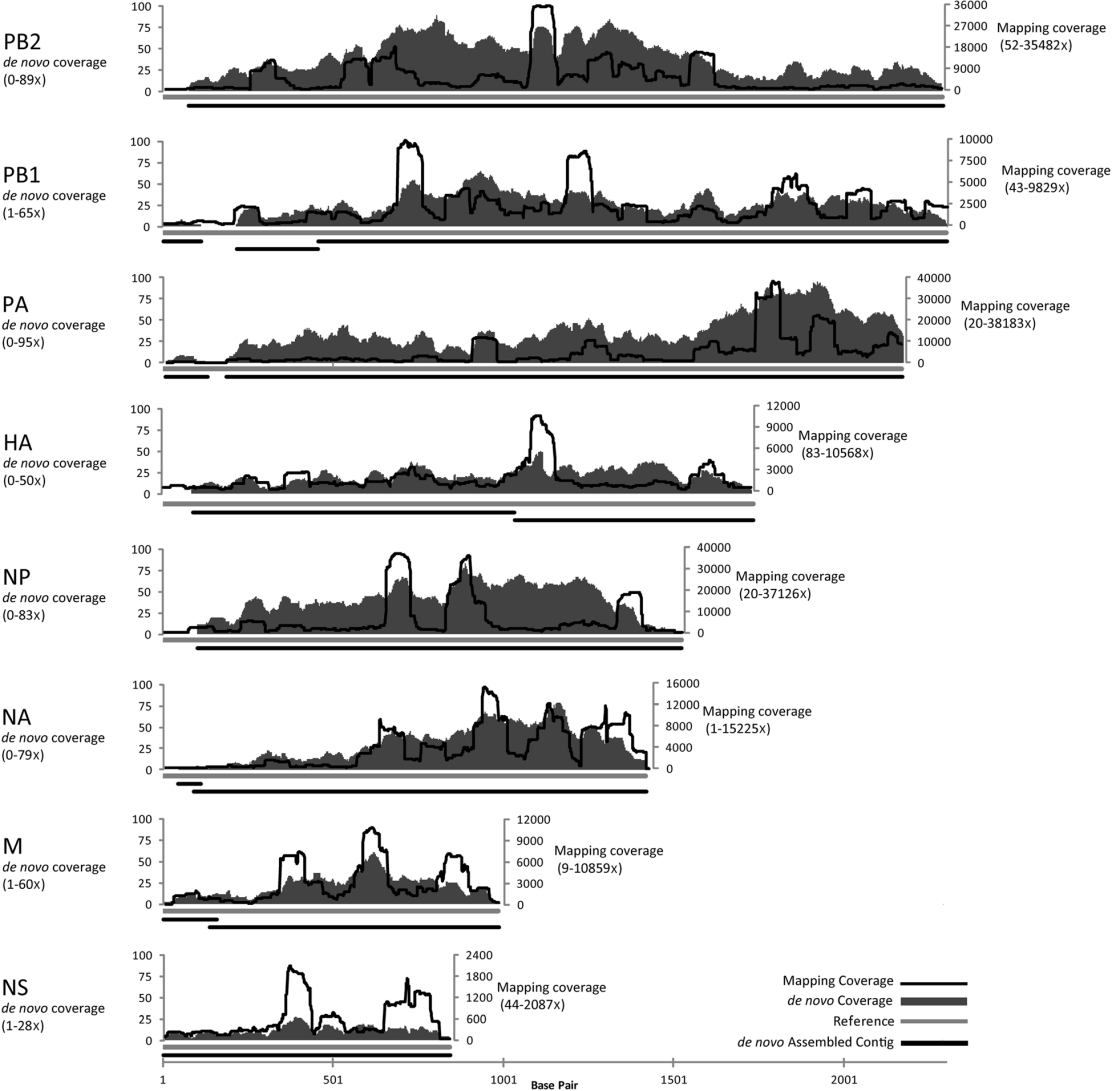
**Mapping and *de novo *assembly coverage sequencing results for the flu SISPA product from a single plaque.** The black lines along the x-axis are the *de novo *assembled contigs mapped to the reference (light gray line across the x-axis). Coverage for the *de novo *contigs is shown with dark gray bars in the graph (left y-axis). The coverage from mapping the reads to the reference is represented with a black line (right y-axis).

In order to determine if this method is capable of producing a complete or nearly complete *de novo* assembled viral genome from a single isolated viral plaque, a variant of the 2009 pandemic H1N1 human Influenza A virus NY1682-MAP7 was plated and a single plaque was subjected to the modified SISPA protocol. The gel-purified SISPA products were sequenced with 454 Titanium and Illumina HiSeq platforms, generating a total of 7,732 Titanium and 1,726,976 HiSeq reads. The sequencing reads were mapped to the reference Influenza genome [GenBank:CY054699-706] and produced a mapping assembly with 3590-fold average coverage representing 100% of the genome. The data was also *de novo* assembled using the Newbler Assembler following the removal of input reads with high sequence identity matches to canine host cells (11.9%), human contaminating sequences (0.7%) and reads containing fragmented SISPA adapter sequences (7.0%). The resulting contigs were mapped to the reference, producing a mapping assembly with 30-fold average sequence coverage, representing 96.5% of the genome (Figure 2). For the segments of PB2, PA, HA, NP, and NA, 6 to 100 bases were missing at the 5′ terminus of each segment, whereas for the segments of PB1, M and NS, the 5' terminal sequences were obtained. A SNP analysis of the *de novo* assembly to the reference sequence revealed 4 SNPs, only 3 resulting in amino acid changes (PA: Val521Ile; NA: Ile185Met; HA: Gly172Glu). However, these SNPs were expected since an Influenza A virus population is known to be a quasispecies, and any single virus from that population may have different sequences compared to the consensus sequence of that population [16]. The *de novo* assembly result of terminal un-covered regions was due to the fact that the SISPA method had relatively lower coverage at the end of linear segments. To add sequence coverage at the genome ends, Djikeng et al. used additional primers specific to the genome ends when performing SISPA [8]; however, adding these sequence-specific primers is not feasible or necessary in this study since the purpose was to modify the SISPA protocol so it can be used to sequence novel viruses where the sequence is unknown.

## Discussion

Before attempting to amplify viral genomes from single isolated plaques, a whole genome amplification method with suitable sensitivity was needed. Whole genome amplification using the Φ29 DNA polymerase (a.k.a. multiple displacement amplification or MDA) requires at least 1 ng of template [17], but a single plaque of bacteriophage λ contains ~0.1 ng of double-stranded DNA (~ 2 × 10^6^ particles) [18]. Another method, Sequence Independent Single Primer Amplification (SISPA) [8,19] has an advantage over MDA in that branching does not occur during amplification. SISPA is also more convenient in that both amplification and fragmentation of the genome are done simultaneously, whereas MDA requires separate time-consuming amplification and fragmentation steps toward the generation of a genomic library for sequencing. SISPA, as modified by Djikeng *et al.,* can routinely amplify between 0.25 and 10 ng of ssRNA and dsDNA templates, respectively [8].

The Klenow reactions of the SISPA method were optimized through more robust removal of host nucleic acids, altered denaturation and annealing conditions, reduced reaction volumes and greater primer concentrations. Host nucleic acids were more thoroughly removed by using RNase T1 and Benzonase^®^ in addition to the standard RNase A and DNase I treatment [8]. RNase T1 combined with RNase A resulted in smaller RNA fragments after digestion than when using RNase A alone (data not shown). Benzonase^®^, a genetically engineered endonuclease that can degrade all forms of DNA and RNA, has been shown to be more effective at digesting DNA than DNase I alone [20]. Taking these additional steps in decontaminating the viral sample of host DNA and RNA allows for increased sensitivity in the subsequent amplification process. The addition of DMSO to the denaturation step was not previously used for SISPA [8]; however, it has been shown to disrupt secondary structure of DNA to achieve higher yields in PCR [21]. DMSO can increase non-specific annealing, which is advantageous for random amplification. A snap cooling step after denaturing the template and a temperature ramp for random primer binding were also found to increase the sensitivity and amount of product generated (data not shown). Finally, amplification reaction volumes were optimized through volume reduction (e.g., 5–10 μl) to allow for the template to be at a higher concentration for specific amplification [22]. The concentration of random hexamer primers was greater than originally used for SISPA [8]. It has been previously shown that increasing the primer concentration in PCR results in greater amplification [23] with the consequence of increased non-specific priming, which again is desirable for random amplification of template.

Despite the addition of multiple nucleases, host and human sequences can still be present, albeit at a relatively low level. When amplifying genomic material from very small quantities, the smallest amount of contaminating nucleic acids can cause problems and can be minimized with good sterile technique. Human contaminating sequences can enter before or during the random priming or the library construction steps. Host genomic material can be shielded from nuclease digestion if bound by protein or membranes. For example, it has been known for many years that histones protect DNA from nuclease digestion [24]. Indeed, we saw more contamination from the canine host genome than from the *Pseudomonas* host genome. These low levels of contamination can be removed informatically through similarity searches.

The PCR step of the SISPA protocol was also improved by adding an additional cleanup step to the Klenow reaction and optimizing PCR for products of a size range more applicable to next-generation sequencing technologies rather than Sanger sequencing. The Klenow products were purified using PEG precipitation [11,25,26] prior to treatment with Exonuclease I to help ensure the amplification would contain minimal background generated from any primers or small fragments from the Klenow reaction. The elongation time of the PCR was decreased in order to shift products to a shorter size range that is more suitable for library creation for 454 and Illumina HiSeq platforms. PCR products were also gel extracted in a lower range (300–850 bp) than previously used for SISPA (500–1000 bp) [8] for the same reason. This purification method also resulted in a more robust yield of PCR products with less loss than column purification methods.

Our SISPA protocol was optimized to be able to amplify the minute amount of nucleic acid in a single isolated viral plaque. Starting with just a single isolated viral plaque is advantageous for those samples that are difficult to propagate in the lab and also saves time as culture scale-up and ultracentrifugation are not required. Additionally, there is less host contamination present in just one viral plaque compared to a large liquid stock, allowing for cleaner downstream analysis. The protocol was optimized using a greater concentration of random hexamer primers than originally used for SISPA without the need for tagged poly-dT or conserved sequence primers [8], enabling this method to have a more universal application. Because genomic sequences may exist that are complimentary to the barcode sequence, which will result in uneven sequence coverage, it may be necessary to use more than one barcode per sample to compensate for any sequencing pile up. These changes have produced a SISPA protocol that is robust enough such that a single viral plaque can provide sequencing data that is acceptable for mapping or *de novo* assembly.

## Conclusions

DNA quantities of as low as 10 picograms were sufficient to span 98% of a bacteriophage genome by *de novo* assembly of 454 and Illumina HiSeq data. This procedure was used successfully to sequence and *de novo* assemble a variant of the 2009 pandemic H1N1 human Influenza A virus [9] from a single viral plaque. The method works with 454 and Illumina HiSeq platforms and should also work well on any amplicon-based sequencing platform, including the third generation PacBio or Ion Torrent sequencing technologies.

## Competing interests

The authors declare that they have no competing interests.

## Authors’ contributions

DEF designed the experiment. JP, GK, and JD performed the experiments. BZ cultured the flu virus and harvested the RNA. DEW provided the NY1682-MAP7 Influenza A virus. JMM provided analysis for the sequencing data. JD processed data and generated the figures for the paper. DEF, BZ, JMM, and JD wrote the paper. All authors read and approved the final manuscript.
